# Outcome of amniotic membrane transplantation in ocular GVHD-related corneal melting: a retrospective case series

**DOI:** 10.3389/fmed.2026.1811327

**Published:** 2026-04-30

**Authors:** Xin-Yu Zhuang, Yao Xu, Zheng-Tai Sun, Yue Xu, Ying-Jie Chen, Xiao-Wen Tang, Feng Chen, Xiao Ma, Xiao-Jing Shi, Xiao-Feng Zhang

**Affiliations:** 1 Department of Ophthalmology, The Fourth Affiliated Hospital of Soochow University, Suzhou, Jiangsu, China; 2 Department of Ophthalmology, The First Affiliated Hospital of Soochow University, Suzhou, China; 3 National Clinical Research Center for Hematologic Diseases, Jiangsu Institute of Hematology, The First Affiliated Hospital of Soochow University, Suzhou, China; 4 Institute of Blood and Marrow Transplantation, Collaborative Innovation Center of Hematology, Soochow University, Suzhou, China; 5 The Affiliated Suzhou Hospital of Nanjing Medical University, Suzhou Municipal Hospital, Suzhou, China

**Keywords:** amniotic membrane transplantation (AMT), corneal melting, dry eye, graft versus-host disease (GVHD), ocular surface

## Abstract

**Purpose:**

To investigate the outcome of single amniotic membrane transplantation (AMT) in the treatment of corneal melting associated with ocular graft-versus-host disease (oGVHD) after allogeneic hematopoietic stem cell transplantation (HSCT), and to explore the correlative risk factors.

**Methods:**

A retrospective case series study. Data of 303 patients with oGVHD were reviewed, and cases of corneal melting undergoing AMT were collected. Patients were divided into the single-AMT success group and the single-AMT failure group according to the recurrence of corneal melting within 6 months after receiving the initial AMT. The preoperative ocular assessments (including corneal fluorescence staining, conjunctival congestion, lid margin lesion score, and corneal optical coherence tomography) and characteristics related to HSCT were compared between the two groups.

**Results:**

A total of 31 patients were enrolled in this retrospective study, comprising 21 males (67.74%). At 6 months after the initial AMT, 21 cases achieved corneal healing, while corneal melting recurred in 10 cases. There were no differences in the primary disease and donor sources between the single-AMT success group and the failure group. The incidence of lung GVHD in the failure group was higher than that in the success group (OR = 8.021, 95% CI: 1.379 to 64.902, *p* = 0.020).

**Conclusion:**

Amniotic membrane transplantation can be used as a treatment for oGVHD-related corneal melting. Lung GVHD may be associated with the failure of single AMT surgery. It is recommended to strengthen postoperative immune regulation and follow-up management.

## 1 Introduction

Hematopoietic stem cell transplantation (HSCT) is currently a common treatment for various hematologic malignancies, with over 30,000 patients worldwide receiving allogeneic HSCT every year. 30–70% of allogeneic transplant recipients develop graft-versus-host disease (GVHD) due to response from donor to host human leukocyte antigen (HLA) and/or small histocompatibility antigen differences despite immunoprophylaxis (1). The incidence of ocular GVHD (oGVHD) after HSCT is about 60–90%, which can involve all ocular structures. Dry eye is the main manifestation of oGVHD, which often impairs the quality of life due to discomfort symptoms and can lead to severe vision loss due to corneal involvement (2, 3).

Corneal melting is a rare blinding complication of oGVHD. The structure of the cornea may deteriorate rapidly and lead to perforation, which can result in severe vision loss and may involve both eyes (4–6). At present, there is still a lack of a standard therapeutic regimen for oGVHD-related corneal melting. The common treatments are mainly empirical, including increasing the moisture content of the ocular surface and reducing ocular inflammation with topical application of eye drops (7). For severe, persistent, and complex corneal injury, surgical treatment is generally required according to the condition (8). Amniotic membrane transplantation (AMT) functions as a biological barrier and a biologically active substrate that regulates immune responses, promotes epithelialization, inhibits inflammation, and reduces subsequent corneal scarring. AMT has been effectively used to treat keratitis, neurotrophic keratopathy, non-healing corneal ulcers, and corneal perforations (9). AMT may be a very valuable method for treating refractory oGVHD (10, 11). However, there are relatively limited reports. By reviewing the data from oGVHD patients who developed corneal melting and underwent AMT, our study proposed the surgical indications for AMT in corneal melting based on corneal fluorescence staining and the stromal involvement. We further analyzed cases of recurrent corneal melting within 6 months after a single AMT surgery to investigate factors influencing postoperative prognosis.

## 2 Materials and methods

### 2.1 Subjects

This was a retrospective case series study conducted in the Ophthalmology Department of the First Affiliated Hospital of Soochow University and the Fourth Affiliated Hospital of Soochow University from March 2021 to August 2025.

In this study, patients who met the following inclusion criteria were enrolled: (1) diagnosed with a hematologic disease at the hematology department and had undergone HSCT at least 3 months prior; (2) with ocular symptoms after HSCT, such as dry eye, photophobia and foreign body sensation, and met the diagnostic criteria for chronic oGVHD defined by National Institutes of Health (NIH) consensus (12). Exclusion criteria: (1) with unstable vital signs; (2) with uncontrolled systemic infections; (3) with previous history of other ocular surface diseases before HSCT; (4) with glaucoma and/or intraocular infections; (5) with previous history of ocular surgery. Patients with corneal melting were selected, and statistical analysis was performed on those who underwent AMT.

This study was approved by the ethics committees of the First Affiliated Hospital of Soochow University (No. 2025/1267) and the Fourth Affiliated Hospital of Soochow University (No. 2025/251292), and adhered to the tenets of the Declaration of Helsinki.

### 2.2 Preoperative baseline data

The baseline data of the patients were reviewed, including age, gender, the interval between HSCT and the first ophthalmology visit, the interval between HSCT and cornea melting, primary disease, donor source, HLA type, ABO-blood type, kinship, gender consistency, source of stem cell, and the history of systemic GVHD.

### 2.3 Ocular surface assessment

Ocular surface evaluations were independently conducted by two physicians (XYZ and YJC) in a masked fashion, with discrepancies adjudicated by a third senior ophthalmologist (XFZ) to establish the final results for analysis. In cases of unilateral corneal melting, the affected eye was analyzed. For bilateral involvement, the more severely affected eye was analyzed.

Corneal lesions were observed with corneal fluorescein staining (CFS). The cornea was divided into five regions: superior, inferior, nasal, temporal, and central, and the involvement of corneal regions was recorded. The proportion of corneal melting area at baseline and after treatment was analyzed using ImageJ (version 1.53c, National Institutes of Health, Bethesda, MD, United States).

Conjunctival congestion was scored, ranging from 0 to 2, using the method described by Ogawa et al. (13). The score of lid margin hyperemia ranged from 0 to 3, while the score of irregularity ranged from 0 to 2, using the method described by Arita et al. (14).

The cross-section of the cornea was scanned using an optical coherence tomography (OCT) device (SPECTRALIS OCT, Heidelberg Engineering GmbH, Heidelberg, Germany) with the anterior segment module. The morphology of the corneal epithelial and corneal stromal layers was observed, and the depth of anterior corneal surface depression caused by corneal melting was marked.

Patients with corneal melting were diagnosed according to the following criteria: (1) CFS showed that corneal lesions fusing into patches; (2) OCT showed the absence of the entire epithelial layer in the corneal lesion area, with/without anterior stromal layer defects.

### 2.4 Amniotic membrane transplantation

**Figure 1:**
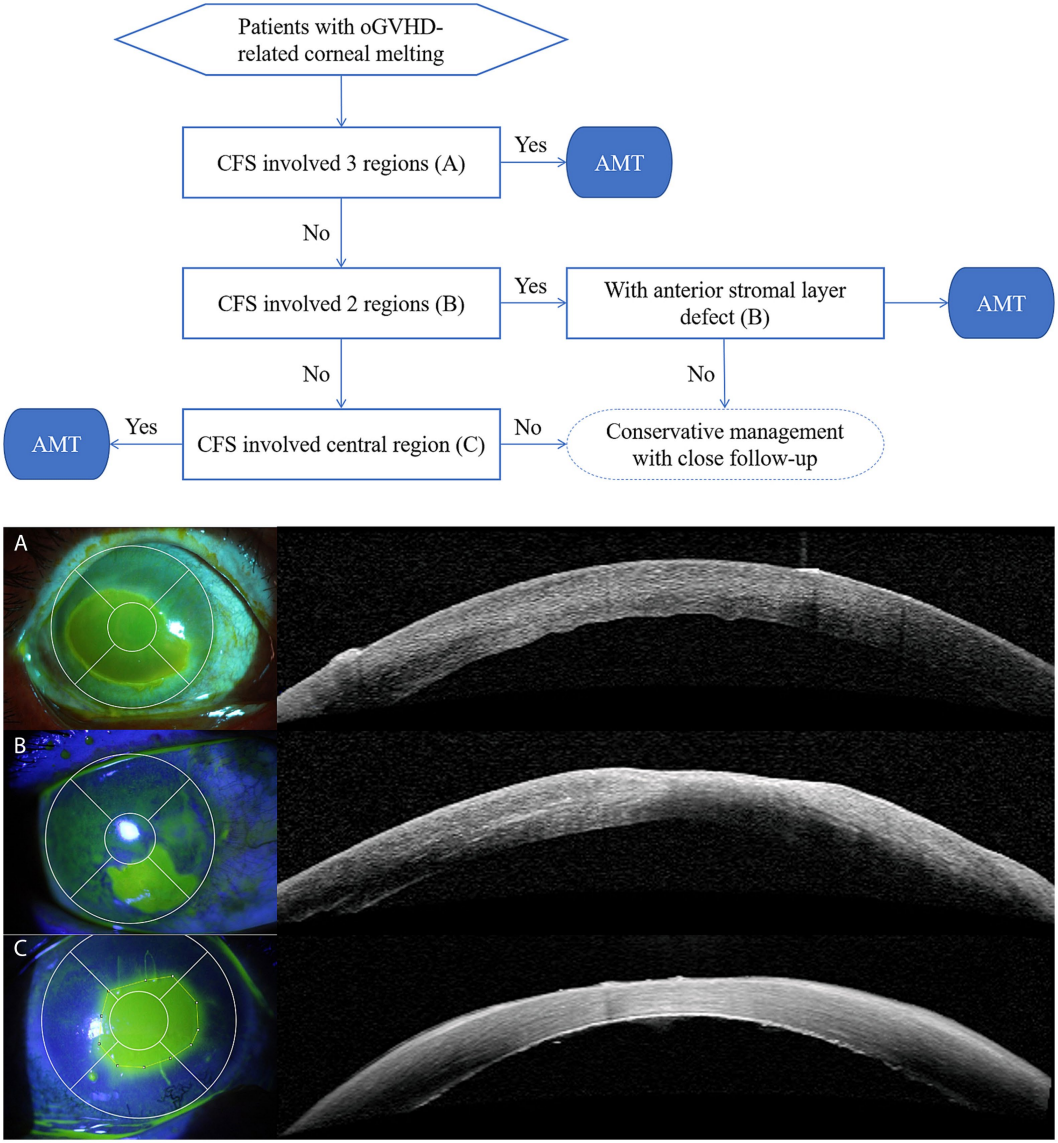
Treatment decision flowchart. **(A)** CFS of corneal lesions involved 5 regions. OCT showed that the corneal epithelial layer in the lesion area was completely absent with no anterior stromal layer defect; **(B)** CFS of corneal lesions involved 2 regions. OCT showed that the corneal epithelial layer in the lesion area was completely absent with anterior stromal layer defect; **(C)** CFS of corneal lesions involved the central region. OCT showed that the corneal epithelial layer in the lesion area was completely absent with anterior stromal layer defect.

Patients who met the following indications were promptly treated with AMT: (1) CFS involved 3 or more regions; (2) CFS involved 2 regions, and OCT showed that the corneal epithelial layer in the lesion area was completely absent with anterior stromal layer defects; (3) CFS involved the central region, and OCT showed that the corneal epithelial layer in the lesion area was completely absent with/without anterior stromal layer defects (Figure 1).

During the surgery, the freeze-dried biological amniotic membrane (AM, Ruiji Bioengineering Technology Co., LTD, China) with 15 mm × 15 mm sized was adopted after rehydration with isotonic saline solution. The AM and bulbar conjunctiva were intermittently sutured to the corneal limbus with 10–0 nylon for fixation, and the stitches were removed 1 week postoperatively.

Topical eye drops were given after AMT, as 0.1% tacrolimus eye drops (Senju Pharmaceutical Co., Ltd.) 4 times a day, 50% autologous serum eye drops 4 times a day, 0.3% ofloxacin eye ointment (Santen Pharmaceutical Co., Ltd.) 3 times a day, and 0.3% sodium hyaluronate eye drops (Santen Pharmaceutical Co., Ltd.) 3 times a day. All subjects received standard hematological treatment according to their individual conditions prior to surgery, and systemic therapy was continued without modification postoperatively.

The prognosis was evaluated based on the CFS area 6 months post-AMT, with success classified as complete resolution of CFS and failure as recurrence of corneal melting. According to the outcomes, the patients were divided into the single-AMT success group and the single-AMT failure group.

### 2.5 Statistical analysis

SPSS software (version 26.0, IBM Corp., New York, United States) and R software (version 4.5.1, R Foundation for Statistical Computing, Vienna, Austria) were used.

Continuous variables were summarized as mean ± standard deviation (SD) or as median (first quartile, third quartile) if the Kolmogorov–Smirnov test showed a non-normal distribution. Comparisons between groups were performed using the independent-samples *t*-test for normally distributed variables and the Mann–Whitney *U* test for non-normally distributed variables. Categorical variables were summarized as frequency (percentage). Chi-square test was used for comparisons, with Fisher’s exact test applied when the expected frequency was less than 5. Variables with *p* < 0.2 in univariable logistic analysis were included in multivariable logistic regression analysis using Firth’s correction. Time to recurrence of corneal melting was recorded, and Kaplan–Meier survival curves with Log-rank test were performed for cases with recurrence based on statistically significant variables. The predictive performance of these significant variables for corneal melting recurrence was assessed using receiver operating characteristic (ROC) curve analysis. A *p*-value of <0.05 was considered statistically significant.

## 3 Results

### 3.1 Demographic information

A total of 303 patients with oGVHD were reviewed, of which 63 patients (20.79%) presented unilateral or bilateral corneal melting. Among these cases, a total of 31 eligible cases (49.21%) received AMT, comprising 21 males (67.74%) and 10 females (32.26%). The mean age of patients was 39.55 ± 11.48 years old. The mean interval between HSCT and first visit to the Ophthalmology Department was 25.87 ± 31.83 months. The mean interval between HSCT and corneal melting was 49.77 ± 38.88 months.

There were 21 eyes (67.74%) in the single-AMT success group, and 10 eyes (32.26%) in the single-AMT failure group. In the single-AMT success group, corneal melting was unilateral in 11 patients (52.38%) and bilateral in 10 patients (47.62%), whereas in the failure group, 3 patients (30.00%) had unilateral and 7 patients (70.00%) had bilateral corneal melting. There was no statistically significant difference in the number of involved eyes between the two groups (*χ*^2^ = 1.370, *p* = 0.280). The average interval between AMT and corneal healing was 24.48 ± 17.15 days. In the failure group, the mean interval between AMT and the recurrence of corneal melting was 57.30 ± 21.74 days.

### 3.2 Clinical characteristics

There were 15 cases of acute myelogenous leukemia (AMT, 48.39%), 5 cases of acute lymphoblastic leukemia (ALL, 16.13%), 3 cases of chronic myelogenous leukemia (CML, 9.68%), 3 cases of myelodysplastic syndrome (MDS, 9.68%), 3 cases of Mixed phenotype acute leukemia (MPAL, 9.68%), and 2 cases of lymphoma (6.45%).

In terms of donor source, 14 cases underwent haploidentical hematopoietic stem cell transplantation (Haplo-HSCT, 45.16%), 14 cases underwent sibling compatible HSCT (Sib-HSCT, 45.16%), and 3 cases underwent unrelated donor HSCT (URD-HSCT, 9.68%). Seventeen cases (54.84%) were HLA-matched, while 14 cases (45.16%) were HLA-mismatched. Twenty-eight cases (90.32%) received grafts from related donors, while 3 cases (9.68%) received grafts from unrelated donors. Sixteen cases (51.61%) had ABO-mismatched donors, while 15 cases (48.39%) had ABO-matched donors. Nineteen cases (61.29%) received gender mismatched transplantation, and 12 cases (38.71%) received gender matched transplantation. Nineteen cases only used peripheral blood stem cells (PBSCT, 61.29%), 2 cases only used bone marrow stem cells (BMT, 6.45%), while 10 cases (32.26%) used both BMT and PBSCT.

Of the 31 patients, 10 cases (96.77%) had organ involvement, and 22 cases (73.33%) of them had rejection in multiple organs, 8 cases (26.67%) had single-organ rejection. The organs involved included skin (22 cases), oral mucosa (17 cases), liver (16 cases), lungs (7 cases), gastrointestinal tract (6 cases), and 1 case each involving the kidney, spleen, muscles, joints, mucosa, and fingernails.

The incidence of lung GVHD between the two groups was significantly different (*p* = 0.022). No significant difference was observed between the two groups in clinical characteristics, including primary diseases and donor sources (Table 1).

**Table 1:** Comparisons of preoperative baseline data between the single-AMT success group and single-AMT failure group.

Parameters	Single-AMT success group	Single-AMT failure group	*p*-value
Gender			
Male	15/21	6/10	0.685
Female	6/21	4/10	
Age (years)			
	39.24 ± 11.88	40.20 ± 11.18	0.832
Interval between HSCT and the first ophthalmology visit (months)			
	17.33 (4.25, 32.53)	15.90 (10.24, 39.69)	0.519
Interval between HSCT and cornea melting (months)			
	43.41 ± 37.82	63.14 ± 39.58	0.191
Primary disease			
AML	10/21	5/10	0.438
ALL	5/21	0/10	
CML	2/21	1/10	
MDS	1/21	2/10	
MPAL	2/21	1/10	
Lymphoma	1/21	1/10	
Donor source			
Haplo-HSCT	10/21	4/10	0.491
Sib-HSCT	10/21	4/10	
URD-HSCT	1/21	2/10	
HLA			
Fully match	11/21	6/10	1.000
Partially match	10/21	4/10	
Kinship			
Related	20/21	8/10	0.237
Unrelated	1/21	2/10	
ABO compatibility			
Matched	9/21	6/10	0.458
Mismatched	12/21	4/10	
Gender relationship			
Matched	9/21	3/10	0.697
Mismatched	12/21	7/10	
Source of stem cell			
BMT	1/21	1/10	0.478
PBSCT	12/21	7/10	
BMT + PBSCT	8/21	2/10	
Systemic GVHD			
With	21/21	9/10	0.323
Without	0/21	1/10	
Number of organs with GVHD			
Single	7/21	1/9	0.374
Multiple	14/21	8/9	
Organs number	2.0 (1.0, 3.0)	3.0 (1.75, 4.0)	0.233
Organs with GVHD			
Skin	14/21	8/10	0.677
Oral mucosa	11/21	6/10	1.000
Liver	11/21	5/10	1.000
Gastrointestinal tract	4/21	2/10	1.000
Lung	2/21	5/10	**0.022**

AMT, amniotic membrane transplantation; HSCT, hematopoietic stem cell transplantation; AML, acute myelogenous leukemia; ALL, acute lymphoblastic leukemia; CML, chronic myelogenous leukemia; MDS, myelodysplastic syndrome; MPAL: Mixed phenotype acute leukemia; Haplo-HSCT, haploidentical hematopoietic stem cell transplantation; Sib-HSCT, sibling compatible hematopoietic stem cell transplantation; URD-HSCT, unrelated matched donor hematopoietic stem cell transplantation; HLA, human leukocyte antigen; BMT, bone marrow transplantation; PBSCT, peripheral blood stem cell transplantation; GVHD, graft-versus-host disease.

Continuous variables conforming to the normal distribution are reported as mean ± standard deviation, while those not normally distributed are reported as median (first quartile, third quartile). Categorical variables are reported as number of cases/total sample size.

The bold value: a *P*-value of <0.05 was considered to be statistically significant.

### 3.3 Preoperative ocular surface assessment

For the total cohort, the mean CFS area proportion for corneal melting was 43.24% ± 22.33%. The median number of regions for corneal melting involving was 5.0(3.5, 5.0). The central pupil region of the cornea was involved in 30 eyes (96.77%), the superior region was involved in 18 eyes (58.06%), the inferior region was involved in 29 eyes (93.55%), the nasal region was involved in 26 cases (83.87%), and the temporal region was involved in 28 cases (90.32%). The superior region was involved significantly less than those in other regions (*χ*^2^ = 22.875, *P* < 0.001). The median preoperative conjunctival congestion score was 2.0 (1.5, 2.0) in 31 eyes. The median preoperative lid margin hyperemia score was 3.0 (2.5, 3.0). The median preoperative lid margin irregularity score was 1.0 (1.0, 2.0). Corneal neovascularization was observed in 11 eyes (35.48%), and 16 eyes (51.61%) had corneal stromal involvement before AMT.

There were no statistically significant differences across all preoperative corneal assessments between the singly-AMT success group and failure group (Table 2).

**Table 2:** Comparisons of preoperative ocular assessment between the single-AMT success group and single-AMT failure group.

Parameters	Single-AMT success group	Single-AMT failure group	*P*-value
Area proportion of CFS			
	43.24% ± 22.33%	40.68% ± 21.18%	0.763
Number of CFS regions			
	5.0 (3.5, 5.0)	4.0 (3.0, 5.0)	0.603
Region involved in corneal melting			
Center	20/21	10/10	1.000
Superior	13/21	5/10	0.701
Inferior	19/21	10/10	1.000
Nasal	18/21	8/10	1.000
Temporal	20/21	8/10	0.237
Corneal neovascularization			
With	8/21	3/10	1.000
Without	13/21	7/10	
Corneal stroma involved			
With	11/21	5/10	1.000
Without	10/21	5/10	
Conjunctival congestion			
	2.0 (1.5, 2.0)	1.5 (1.0, 2.0)	0.250
Lid margin hyperemia			
	3.0 (2.5, 3.0)	3.0 (2.0, 3.0)	0.852
Lid margin irregularity			
	1.0 (1.0, 2.0)	1.0 (0.0, 2.0)	0.633

AMT: amniotic membrane transplantation; CFS: corneal fluorescence staining Continuous variables conforming to the normal distribution are reported as mean ± standard deviation, while those not normally distributed are reported as median (first quartile, third quartile).Categorical variables are reported as number of cases/total sample size.

### 3.4 Factors associated with single-AMT failure

In the univariable logistic regression analysis, lung GVHD was significantly associated with corneal melting recurrence (OR = 7.800, 95% CI: 1.441 to 54.766, *p* = 0.017). After adjusting for the interval between HSCT and corneal melting, multivariable logistic regression analysis revealed that lung GVHD remained significantly associated with single-AMT failure (OR = 8.021, 95% CI: 1.379 to 64.902, *p* = 0.020), indicating that patients with lung GVHD had 8.021-fold higher odds of AMT failure compared to those without lung GVHD (Table 3).

**Table 3:** Univariable and multivariable logistic regression analysis for factors associated with single-AMT failure.

Factors	Univariable regression analysis		Multivariable regression analysis	
	OR (95%CI)	*P*-value	OR (95%CI)	*P*-value
Demographics				
Age (years)	1.007 (0.944–1.075)	0.837		
Gender	1.651 (0.352–7.610)	0.517		
Clinical characters				
Interval between HSCT and the first ophthalmology visit (months)	1.008 (0.986–1.031)	0.457		
Interval between HSCT and cornea melting (months)	1.012 (0.994–1.033)	0.194	1.015 (0.994–1.039)	0.152
Primary disease	1.201 (0.752–1.927)	0.437		
Donor source	1.641 (0.547–5.221)	0.373		
HLA	0.758 (0.166–3.261)	0.710		
Kinship	4.020 (0.465–49.032)	0.201		
ABO compatibility	0.526 (0.114–2.256)	0.388		
Gender relationship	1.629 (0.368–8.256)	0.526		
Source of stem cell	0.515 (0.123–1.836)	0.308		
History of systemic GVHD	0.147 (0.001–3.021)	0.213		
Number of organs with GVHD	1.405 (0.803–2.590)	0.234		
Skin GVHD	1.759 (0.359–11.153)	0.497		
Oral GVHD	1.319 (0.307–6.016)	0.71		
Liver GVHD	0.913 (0.210–3.962)	0.902		
Gastrointestinal tract GVHD	1.144 (0.171–6.405)	0.881		
Lung GVHD	7.800 (1.441–54.766)	**0.017**	8.021(1.379–64.902)	**0.020**
Ocular surface assessment				
Area proportion of CFS	0.616 (0.018–17.994)	0.778		
Number of CFS regions	0.775 (0.322–1.855)	0.562		
Central region involved	1.537 (0.075–232.300)	0.792		
Superior region involved	0.630 (0.142–2.747)	0.534		
Inferior region involved	2.692 (0.194–384.962)	0.498		
Nasal region involved	0.643 (0.103–4.490)	0.637		
Temporal region involved	0.249 (0.020–2.150)	0.201		
Corneal neovascularization	0.741 (0.146–3.328)	0.700		
Corneal stroma involved	0.913 (0.210–3.962)	0.902		
Conjunctival congestion	0.333 (0.069–1.522)	0.155	0.362 (0.06–1.935)	0.233
Lid margin hyperemia	0.900 (0.243–3.859)	0.874		
Lid margin irregularity	0.732 (0.270–1.914)	0.521		

AMT, amniotic membrane transplantation; HSCT, hematopoietic stem cell transplantation; AML, acute myelogenous leukemia; ALL, acute lymphoblastic leukemia; CML, chronic myelogenous leukemia; MDS, myelodysplastic syndrome; MPAL: Mixed phenotype acute leukemia; Haplo-HSCT, haploidentical hematopoietic stem cell transplantation; Sib-HSCT, sibling compatible hematopoietic stem cell transplantation; URD-HSCT, unrelated matched donor hematopoietic stem cell transplantation; HLA, human leukocyte antigen; BMT, bone marrow transplantation; PBSCT, peripheral blood stem cell transplantation; GVHD, graft-versus-host disease; CFS: corneal fluorescence staining.

The bold value: a *P*-value of <0.05 was considered to be statistically significant.

**Figure 2:**
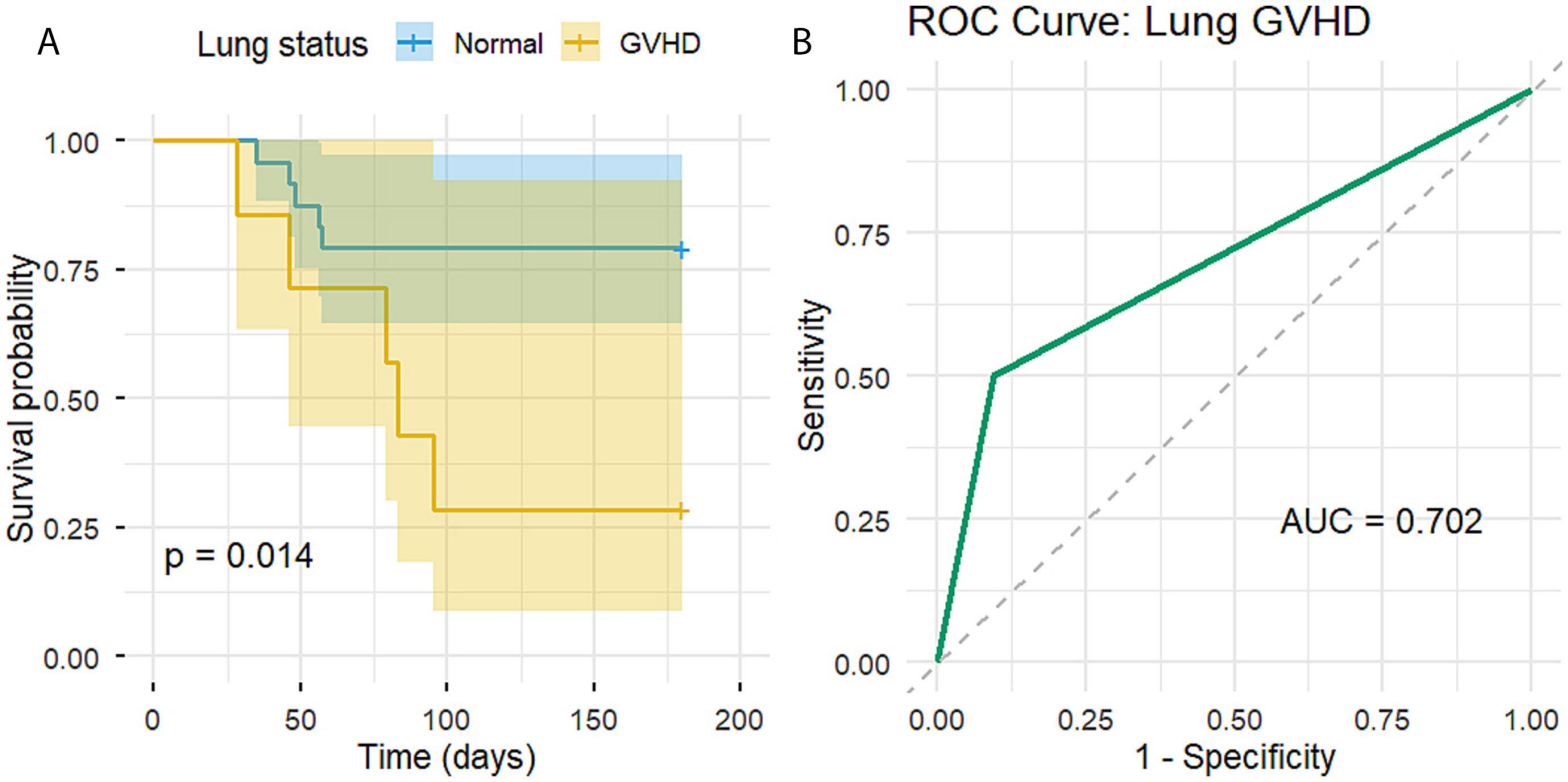
Association of lung GVHD with recurrent corneal melting after AMT. Kaplan-Meier survival curve **(A)**. Patients were grouped according to the presence of lung GVHD to assess the probability and timing of recurrent corneal melting after the initial AMT. Blue line represents eyes without lung GVHD, and red line represents eyes with lung GVHD. ROC curve analysis **(B)**. Lung GVHD demonstrated the predictive ability for recurrent corneal melting after a single AMT, with an AUC of 0.702 (95% CI 0.527 to 0.878), sensitivity of 50.0%, and specificity of 90.5%.

After 6 months of follow-up, the 31 patients were further divided into two groups based on the presence of lung GVHD, and the difference in survival was compared (Figure 2). The curve of the non-lung GVHD group (24 cases) declined more slowly, while that of the lung GVHD group (7 cases) declined more steeply. The Log-rank test showed that, given the sample size, lung GVHD had a statistically significant impact on the risk of recurrent corneal melting after a single AMT (*χ*^2^ = 6.082, *p* = 0.014). Patients with lung GVHD were more likely to experience failure after a single AMT.

ROC curve analysis showed that lung GVHD was predictive of single AMT outcome, with an area under the curve (AUC) of 0.702 (95% CI 0.527 to 0.878), sensitivity of 50.0%, and specificity of 90.5% (Figure 2).

## 4 Discussion

In this study, we described the clinical features of oGVHD-related corneal melting and observed that the success rate of single-AMT was 67.74% after 6-month follow-up. After further analyses between the single-AMT success and failure groups, lung GVHD was observed to be associated with the risk of corneal melting recurrence after a single AMT.

During the pathogenesis of oGVHD, the complex imbalance of the immune system constitutes the core role in the disease progression. Due to its low immunogenicity, the AM is generally well tolerated, with minimal risk of rejection or serious postoperative complications, and its immunological safety and favorable clinical tolerance have been documented (15–17). AM is also rich in cytokines, growth factors and protease inhibitors, and has anti-fibrotic, anti-inflammatory and anti-angiogenic properties (18, 19). AMT can be performed in patients with persistent epithelial defects (20). At present, there have been relevant case reports on the application of AM in the surgical management of severe corneal lesions related to oGVHD (10, 21–26). In addition to its direct benefits for healing severe corneal ulcers and potentially preventing perforation, AMT can also prevent the recurrence of severe oGVHD, which is related to the potential immunomodulatory properties of the AM (10). Patch AMT can modulate inflammation, inhibit proteolytic activity, and prevent further tissue damage, making it an ideal early intervention to suppress inflammation and promote tissue regeneration (19). However, the optimal timing and surgical indications for AMT in oGVHD-related corneal melting treatment still lack quantitative stratification, and the postoperative prognosis after AMT varies considerably.

**Figure 3:**
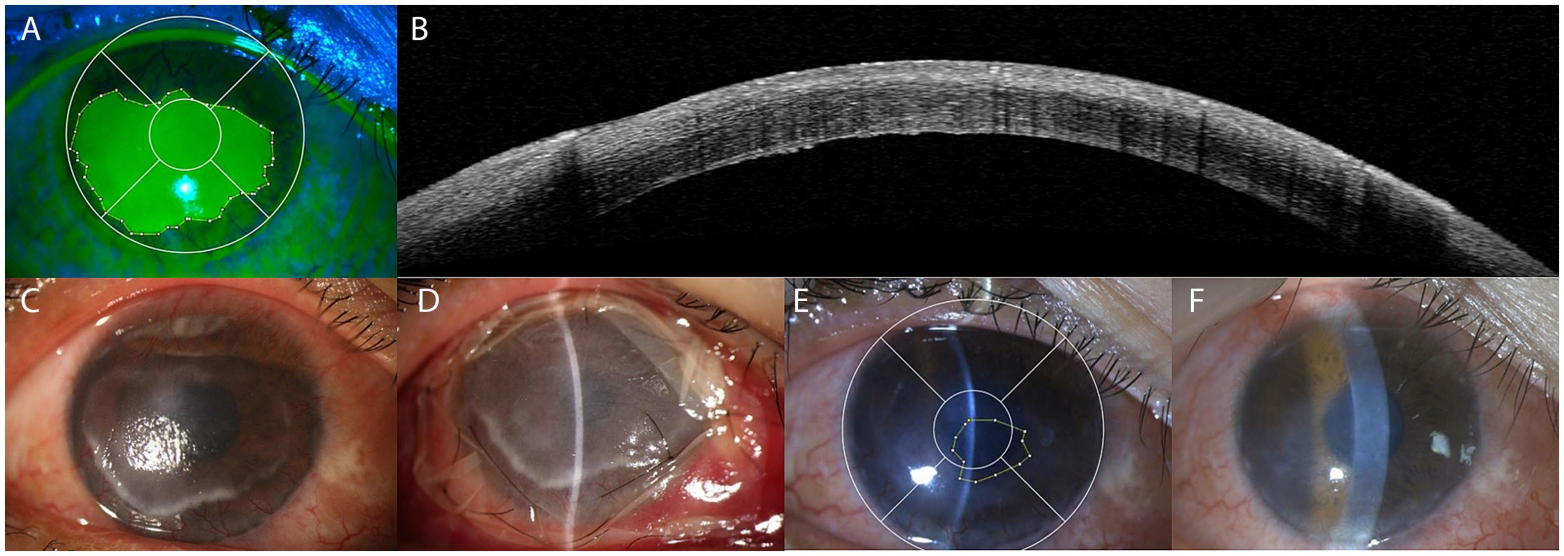
Case of AMT treatment in oGVHD-related corneal melting. A 30-year-old male, 3 years after sibling HSCT for CML (ABO-mismatched donor; combined BMT and PBSCT), presented with skin and oral GVHD. Corneal melting occurred in his right eye with a CFS area proportion of 50.67%, and 5 regions involved **(A)**. The slit lamp examination showed the neovascularization in the whole corneal limbus, conjunctival congestion, lid margin hyperemia and slightly irregularity **(C)**. The OCT examination showed the absence of the full epithelial of cornea **(B)**. AMT was performed **(D)**. The AM was removed 1 week later, and the corneal lesion improved, with only approximately 5.78% of the epithelial defect remaining unhealed **(E)**. The corneal epithelium healed after subsequent conservative treatment, with no recurrent corneal melting observed during the 6-month follow-up **(F)**.

Among the patients with corneal melting enrolled in this study, 21 eyes achieved corneal healing after a single AMT (Figure 3). In the remaining 10 patients, corneal melting recurred during follow-up despite initial healing or improvement after AMT. This study compared the system GVHD status between the two groups. Lung GVHD was observed in 2 cases (9.52%) in the success group, compared with 50% in the failure group. The proportion of patients with skin GVHD was also higher in the failure group (80%) than in the success group (66.67%). The correlation between systemic GVHD and the occurrence of oGVHD has been reported, and a history of acute GVHD is considered a risk factor for the occurrence and development of chronic GVHD (27, 28). The number of organs involved in chronic GVHD has been reported to be associated with the occurrence of oGVHD, particularly in patients with involvement of the oral mucosa, skin, liver, gastrointestinal tract, and lungs (29). Our previous study observed that systemic GVHD was correlated with corneal epithelial lesions, lid margin lesions, and meibomian gland loss, and patients with a history of systemic GVHD were more likely to develop corneal epithelial lesions, especially those with oral and liver GVHD (30). The epidermis and structures most affected in oGVHD (such as lacrimal glands, eyelid epidermis, meibomian glands, cornea, and conjunctiva) originate from the ectoderm, sharing the same embryonic origin as nails and oral mucosa, potentially leading to concurrent involvement of the eyes, skin, and oral cavity (31, 32). Lung GVHD demonstrated a moderate predictive value (AUC = 0.702) with relatively wide confidence intervals indicating a degree of uncertainty, which may be related to the small sample size and heterogeneity of the cohort. Despite these limitations, the identification of lung GVHD as a relevant factor of AMT failure in this study suggests that systemic immune dysregulation may play a critical role in corneal healing. Patients with severe corneal involvement were also noted to have a history of lung GVHD in previous surgical case reports (10, 21, 24, 33). The irreversible fibrosis impairs the physical functioning of the eye, as well as the skin and lungs, through similar underlying pathophysiological mechanisms (34). The lacrimal glands, meibomian glands, salivary glands, hepatic ducts, and pulmonary ducts all belong to the ductal regions, whose surfaces are composed of mucosa and serve as targets for T cells and other inflammatory cells (29). Inflammatory factors such as IL-6 and TNF-α are simultaneously activated in both the ocular surface and pulmonary tissue, which may explain the adverse effect of systemic GVHD on corneal healing. The progression of oGVHD is closely related to systemic GVHD activity, and chronic GVHD may persist in the target organs even after systemic therapy (23). Particularly in patients with lung GVHD, diagnosis may be delayed due to stringent diagnostic criteria (35). This condition may often be associated with more severe and persistent systemic inflammation, with the immune activation state extending beyond the pulmonary system. Consequently, corneal lesions tend to be prolonged, recurrent, progressive, and refractory, often requiring multiple AMT interventions. Therefore, oGVHD management should integrate systemic GVHD assessment and immunologic control. In this context, AMT can be regarded as an effective local therapeutic intervention targeting ocular surface pathology.

Previous studies have identified several factors related to the development of oGVHD, including recipient age, primary disease type, donor source, HLA type, ABO-blood type, kinship, gender consistency, and source of stem cell (28, 36–40). In the present study, we also evaluated these variables; however, no statistically significant associations were observed. Given the rarity of oGVHD-related corneal melting, we included as many eligible subjects as possible, despite substantial heterogeneity within the cohort. Although multivariable regression analyses were performed to account for potential confounding factors, such heterogeneity may have contributed to the observed variability in outcomes and obscured potential between-group differences. Consequently, some associations may not have reached statistical significance despite underlying biological relevance.

Reviewing the cases, the number of eyes experiencing recurrent corneal melting increased in the single-AMT failure group from 50 days postoperatively. Considering that the cellular junctions of the healing corneal epithelium were not sufficiently tight, they were prone to be disturbed by external factors, causing the recurrence of corneal defects and melting. Therefore, irregular follow-up may delay timely treatment adjustments in response to disease progression. Optimizing follow-up frequency and intervention strategies may be essential to enhance corneal healing and reduce recurrence.

The exact pathological mechanism of corneal melting and perforation in oGVHD remains to be clarified. Keratoconjunctivitis sicca, the most common symptom of oGVHD, can cause spot-like corneal epithelial lesions, painful corneal epithelial erosion, and filamentous keratopathy, and may progress to further complications such as secondary infection, non-healing corneal ulcers, stromal thinning and necrosis, and even corneal perforation (33, 41). Our previous studies found that 3.5% of patients with oGVHD suffered corneal melting or perforation (6). The severity of corneal lesions in oGVHD was closely related to lid margin lesions, with higher lid margin lesion scores reflecting more severe corneal pathology. Skin GVHD combined with severe lid margin lesions can be a risk factor for corneal melting and perforation (40). In the present study, all 31 eyes that underwent AMT exhibited marked lid margin hyperemia. Eyelid changes can lead to ocular damage, including persistent epithelial defects, infectious keratitis, and corneal scarring (42). Previous studies have suggested that topical or systemic use of corticosteroids, as well as topical use of non-steroidal anti-inflammatory drugs, may be associated with corneal ulcers and perforations in patients with oGVHD (43). It has been reported that corneal perforation in GVHD is most likely due to inflammation-induced corneal melting (44). Local inflammation and the overexpression of inflammatory mediators, which are similar to the mechanisms of autoimmune diseases (such as rheumatoid arthritis and arthritis), may play a major role in corneal melting (43, 45). Excessive proteolytic enzyme activity, especially from matrix metalloproteinases and neutrophil elastase, can degrade the corneal stroma, leading to corneal thinning and perforation (19). Studies have shown that the levels of inflammatory cytokines, IL-1, IL-6, IL-8, and TNF-a, in tear film and conjunctival epithelium of patients with dry eye are elevated (46, 47). Disruption of normal tear secretion function can exacerbate corneal ulcers by damaging the ocular surface epithelium. Persistent epithelial defects can cause inflammatory cell infiltration in the corneal stroma, while stimulation of collagen production by corneal fibroblasts or corneal stromal cells may further promote stromal degeneration (48). Therefore, corneal melting and perforation associated with oGVHD may be multifactorial, driven by severe lid margin lesions, skin rejection, dry eye, and persistent epithelial defects. Continuous local inflammation, abnormal expression of cytokines and proteolytic enzymes, and the application of topical or systemic immunosuppressants may all contribute to corneal tissue destruction.

This study has several limitations. First, its retrospective design and relatively limited sample size reflect the low incidence of oGVHD-related corneal melting and the stringent inclusion criteria requiring AMT. Nevertheless, to our knowledge, this study represents the largest two-center cohort to date, specifically focusing on oGVHD-related corneal melting treated with a standardized single AMT strategy, enabling a focused analysis of postoperative outcomes and risk factors. Second, the retrospective nature may introduce selection bias and limit causal inference. However, this design reflects real-world clinical practice, capturing heterogeneous systemic GVHD manifestations, individualized immunosuppressive regimens, and long-term disease evolution that are difficult to replicate in prospective studies. Importantly, all patients were followed longitudinally after AMT, allowing assessment of clinically meaningful recurrence over an extended period. Third, the present study primarily focused on structural restoration, and future studies will further evaluate functional recovery. Finally, inflammatory biomarkers in tear fluid or serum were not analyzed, and further prospective studies with larger cohorts and molecular-level analyses are warranted to validate and biologically contextualize these findings.

The results of this study suggest that oGVHD-related corneal melting requires more proactive management, and AMT should not be considered merely a topical repair. Quantitative stratification based on CFS extent and stromal involvement on OCT guides clinical decision-making, enabling timely AMT intervention to prevent complications such as corneal perforation. In patients with lung GVHD, corneal healing following AMT may be impaired, with an increased risk of recurrent melting, indicating that systemic inflammation may compromise corneal regenerative and reparative capacity, potentially necessitating repeat surgery. Therefore, early systemic immunomodulatory therapy should be prioritized, with AMT serving as an adjunctive local intervention to stabilize basal immune status, improve the local microenvironment, and enhance surgical success for patients with systemic GVHD manifestations. Close and prolonged postoperative follow-up is essential to monitor AM retention and local inflammation, allowing timely secondary interventions. This integrated treatment may improve corneal healing rate, visual prognosis, and overall quality of life significantly.

## Data Availability

The raw data supporting the conclusions of this article will be made available by the authors, without undue reservation.
